# The influence of implant survival on cost-effectiveness in total knee arthroplasty: a patient-level discrete-event simulation study

**DOI:** 10.1186/s13561-026-00822-z

**Published:** 2026-07-17

**Authors:** Pablo Oscar Roza Miguel, Jorge Albareda Albareda, Nuria García-Rodríguez, Eduardo González Fidalgo, Daniel Hernández Vaquero, José Antonio Queiruga Dios, María Rabanal Rubio, Carlos Revenga Giertych, Fernando J Tavares Sánchez-Monge, Carlos Martín Hernández

**Affiliations:** 1 MBA Institute Chair for Medical and Biomechanical Research, University of Oviedo, Gijón, Spain; 2 University of Zaragoza, Zaragoza, Spain; 3 Associate Professor of Marketing and Market Research, University of Oviedo, Oviedo, Spain; 4 Full Professor of Business Organization, University of Oviedo, Oviedo, Spain; 5 University of Oviedo, Oviedo, Spain; 6 Complejo Asistencial Universitario de Salamanca, Salamanca, Spain; 7 Hospital San Juan Grande, Cádiz, Spain; 8 Hospital Clínico Universitario de Valladolid, Valladolid, Spain; 9 Hospital Universitario Miguel Servet - IIS Aragón, Zaragoza, Spain

**Keywords:** Cost-utility analysis, Total knee arthroplasty, Quality-adjusted life years, Discrete-event simulation, Revision surgery, Budget impact analysis, Health-related quality of life, Arthroplasty registry, Prosthetic survivorship, Health economics

## Abstract

**Background:**

Although several studies have evaluated the cost-effectiveness of total knee arthroplasty (TKA) compared with conservative treatment, few have explored how implant revision rates may influence its long-term cost-effectiveness. The objective of this study was to evaluate the impact of implant revision rates on quality of life and cost-effectiveness in patients undergoing TKA.

**Methods:**

A cost-utility analysis from a healthcare system perspective was conducted using an individualized discrete-event simulation model parameterized with data from a Spanish cohort of patients undergoing TKA. Quality-adjusted life years were calculated with the SF-6D index, and implant alternatives were compared by assigning age- and sex-specific revision rates to each simulated patient while projecting long-term quality of life and life expectancy according to general population standards. The prosthetic models compared were those associated with the highest (WorstKnee) and lowest (BestKnee) revision rates in the UK National Joint Registry. The primary outcome was the incremental cost-effectiveness ratio. Secondary analyses included budget impact and deterministic sensitivity analyses.

**Results:**

The projected quality-adjusted life expectancy over 15 years was 9.767 QALYs for WorstKnee, compared with 10.004 QALYs for BestKnee. The incremental cost-effectiveness ratio was €6,219/QALY, which remained below the cost-effectiveness thresholds commonly used in Spain. Use of BestKnee was associated with an additional cost of €332.3 M over 15 years; however, it also led to a substantial reduction in revision surgeries, with 27,878 fewer procedures performed, resulting in gradual cost savings over time.

**Conclusion:**

Within the price ranges currently observed in the Spanish National Health System, total knee prostheses associated with lower revision rates provide a favorable long-term cost-effectiveness profile and may reduce future revision burden despite their higher acquisition cost.

**Supplementary Information:**

The online version contains supplementary material available at https://doi.org/10.1186/s13561-026-00822-z.

## Introduction

Knee osteoarthritis is characterized by severe pain, joint stiffness and limited range of motion, which negatively impacts patients’ quality of life [1]. Total knee arthroplasty (TKA) is considered the treatment of choice for severe cases that do not respond to conservative treatment [2, 3] because of its proven clinical outcomes and cost-effectiveness [1, 4].

In 2020, osteoarthritis affected 595 million individuals globally, with knee osteoarthritis representing the most prevalent subtype [5]. The number of individuals affected is projected to increase by 75% by 2050 [5], thereby increasing the demand for TKA by up to 139% by 2040 in the Medicare population [6]. Excluding child deliveries, TKA is the most frequently performed surgical procedure in the United States [7], and osteoarthritis is associated with the highest expenditure among Medicare beneficiaries aged 65 years or older [8]. In Spain, 39,487 TKAs were performed within the National Health System in 2022, with a mean hospital stay of 4.4 days and a per-procedure cost of €7,318 [9].

In this context, arthroplasties have become a key target for healthcare optimization strategies [10, 11]. Although lower-priced implants may reduce short-term costs, differences in implant survivorship may substantially influence long-term outcomes and revision burden [10, 11]. Despite the overall effectiveness of TKA and its perception as a standardized procedure, up to 21% of patients remain dissatisfied or require early revision surgery [12, 13].

The prosthetic model used in TKA is one of the factors that most directly influences long-term outcomes [14], and revision surgery is considered the main indicator of implant performance [15]. Consequently, several national arthroplasty registries have implemented mechanisms for the early identification of prostheses associated with higher revision rates [16, 17]. However, even implants with a good track record may require revision surgery during the patient’s lifetime [2, 18]. Importantly, revision procedures are more complex, more expensive, and often associated with worse outcomes than primary surgery [2, 19].

Because healthcare systems aim to maximize health outcomes while controlling costs, economic evaluations should consider both implant price and long-term clinical outcomes [20]. In many cases, reducing future healthcare expenditure may require greater initial investment, making longitudinal assessment essential for evaluating treatment value [20].

Economic evaluation plays an important role in healthcare decision-making, particularly in publicly funded systems such as the Spanish National Health System. Although several studies have evaluated the cost-effectiveness of TKA compared with conservative management for knee osteoarthritis [4, 10, 21, 22], only one study has compared the cost-effectiveness of different prosthetic options [23]. Moreover, long-term analyses integrating implant revision rates, age- and sex-adjusted HRQoL trajectories, and individualized simulation models remain scarce. To our knowledge, this is the first Spanish study to use a patient-level discrete-event simulation model based on real-world SF-6D data to evaluate the long-term cost-effectiveness of different TKA implant survivorship profiles.

The aim of this study was to evaluate the impact of implant revision rates on quality-adjusted life expectancy after TKA and, subsequently, determine the cost-effectiveness relationship between different prosthetic models. We hypothesized that implants associated with lower revision rates would be more cost-effective than implants with higher revision rates, despite their potentially higher acquisition cost.

## Materials and methods

### Objectives

The primary objective of the study was to evaluate the incremental cost-effectiveness ratio (ICER) associated with the use of different TKA implant survivorship profiles.

Secondary objectives included estimation of quality-adjusted life expectancy (QALE), revision burden, operating room utilization, and budget impact over a 15-year simulation horizon.

### Target population

The target population consisted of patients of both sexes over the age of 18 undergoing primary TKA as treatment for knee osteoarthritis. TKA is designated by code 81.54 in the International Classification of Diseases, Ninth Revision, Clinical Modification (ICD-9-CM) and by codes 0SRC and 0SRD in the International Classification of Diseases, Tenth Revision, Procedure Coding System (ICD-10-PCS). No other exclusion criteria were applied.

### Rationale of the model

**Fig. 1:**
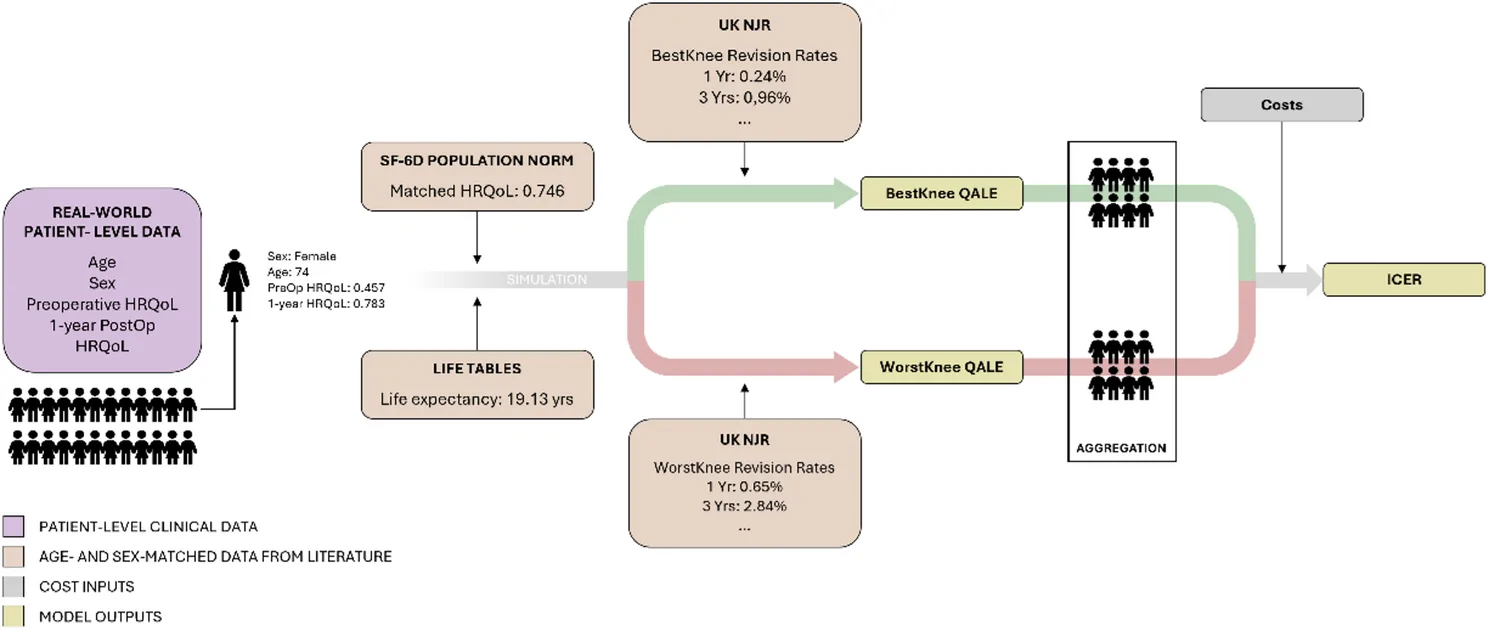
Overall rationale of the model. Real-world patient-level clinical data (age, sex, preoperative and postoperative HRQoL) were combined with age- and sex-specific parameters obtained from the literature, including population HRQoL norms, life expectancy tables, and implant-specific revision rates from the UK National Joint Registry. These parameters were incorporated into an individualized discrete-event simulation model to project long-term quality-adjusted life expectancy (QALE) under two alternative implant survivorship profiles (BestKnee and WorstKnee). Aggregated QALE estimates and implant costs were subsequently used to calculate the incremental cost-effectiveness ratio (ICER)

**Fig. 2:**
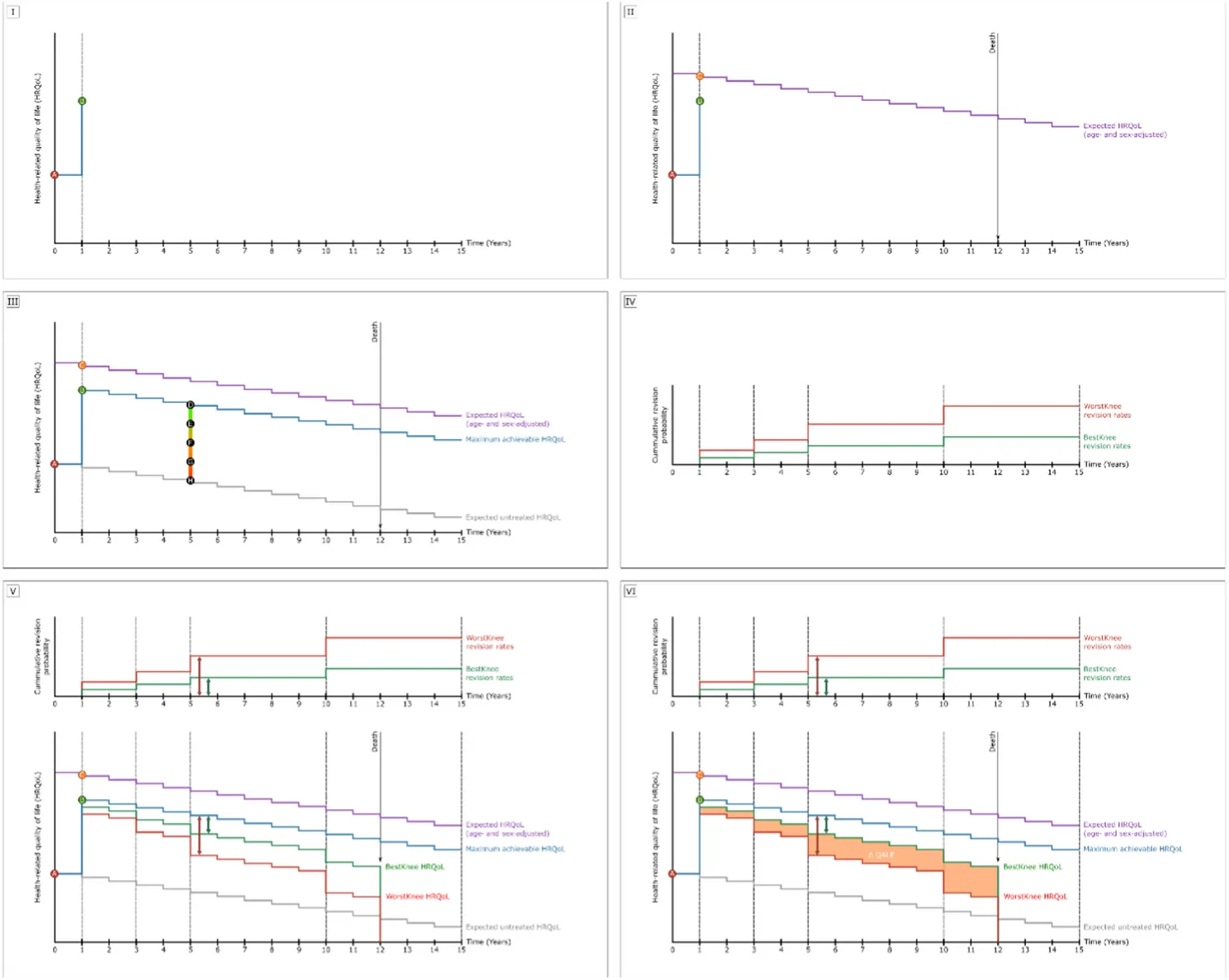
Patient-level simulation example. Slopes were exaggerated for clarity. I: Patient’s HRQoL improves from A (preoperative) to B (1-year postop). II: C represents the age- and sex-matched population HRQoL. Patient life expectancy is estimated from life tables (Death arrow). III: Without implant failure, projected HRQoL follows the blue line (Maximum achievable HRQoL). Without surgery, projected HRQoL follows the grey line (Expected untreated HRQoL). For example, at 5 years after surgery, a 0% revision probability would place the patient at point D, while progressively higher revision probabilities would shift the patient through points E–G, reaching point H if revision probability were 100%. IV: Cumulative revision probabilities for BestKnee and WorstKnee. V: Implant-specific revision rates are applied to the Maximum achievable HRQoL trajectory and constrained by patient life expectancy, generating projected HRQoL trajectories for BestKnee (green) and WorstKnee (red). VI: The orange area represents the incremental gain in quality-adjusted life expectancy

The study aimed to develop a cost-utility analysis, measuring health outcomes in terms of quality-adjusted life years (QALYs) [24, 25]. The analysis was conducted from the perspective of the healthcare system and it was based on a discrete-event simulation developed to predict the evolution of health-related quality of life (HRQoL) in patients undergoing TKA over a 15-year time horizon. The overall rationale of the model is presented in Fig. 1, while Fig. 2 provides a patient-level example illustrating how the model simulates the evolution of HRQoL over time.

To mimic routine clinical practice, we used real-world data from 486 patients belonging to one of the largest clinical series in Spain. For each patient, data were available on age, sex, preoperative HRQoL, and HRQoL one year after surgery.

Previous clinical studies have shown that functional recovery after TKA is achieved within the first postoperative year [3, 26]. Therefore, conventional economic models often assume that HRQoL remains stable thereafter and simply apply a fixed annual discount rate (typically 3% to 5%). Our model follows a different approach. Because age, sex, and baseline HRQoL were available for every patient, each individual’s HRQoL could be compared with the age- and sex-specific HRQoL norms of the general population [27]. Future HRQoL was then projected by applying the age- and sex-specific decline observed in population norms, while preserving the patient’s own starting HRQoL level.

In practical terms, the model assumes that each patient’s HRQoL declines over time following the same age- and sex-specific trajectory observed in the general population, while preserving the patient’s own baseline HRQoL level. In other words, the intercept represents the baseline HRQoL of the patients and the slope reflects the mean trajectory observed in individuals of the same age and sex in the general population.

Similarly, age and sex were used to estimate each patient’s life expectancy using Spanish life Table [28]. After death, utility was assumed to be zero.

However, this projected utility trajectory assumes that the knee implant continues to function indefinitely, which is not clinically realistic. Knee prostheses have an increasing risk of failure over time, and the UK National Joint Registry (NJR) [29] provides revision rates stratified by age and sex. These revision probabilities were applied individually to each patient to simulate the additional loss of utility associated with prosthetic failure.

This framework allows direct comparison between implants with different survivorship profiles. In this study, we projected the quality-adjusted life expectancy that each patient in the cohort would experience if treated with the implant showing the lowest revision rates reported in the UK NJR (BestKnee) versus the implant with the highest revision rates (WorstKnee). Their revision rates, stratified by age and sex are presented in Table 1.

**Table 1:** Revision rates used for the model (adjusted for age and sex)

Prosthetic model	Sex	Age	1 year	3 years	5 years	10 years	15 years
BestKnee	Men	< 55	0.64%	2.64%	3.60%	5.91%	7.25%
		55–64	0.46%	1.64%	2.27%	3.46%	4.22%
		65–74	0.34%	1.11%	1.47%	2.18%	2.51%
		>=75	0.28%	0.76%	0.95%	1.33%	1.39%
	Women	< 55	0.46%	2.31%	3.44%	5.78%	7.03%
		55–64	0.30%	1.43%	2.05%	3.29%	3.90%
		65–74	0.24%	0.96%	1.34%	2.04%	2.29%
		>=75	0.25%	0.69%	0.90%	1.28%	1.36%
	Total		0.32%	1.16%	1.59%	2.44%	2.83%
WorstKnee	Men	< 55	1.74%	7.85%	11.08%	19.51%	29.23%
		55–64	1.24%	4.86%	6.98%	11.43%	17.01%
		65–74	0.92%	3.30%	4.52%	7.18%	10.11%
		>=75	0.77%	2.27%	2.92%	4.38%	5.61%
	Women	< 55	1.24%	6.86%	10.59%	19.07%	28.34%
		55–64	0.82%	4.27%	6.33%	10.86%	15.74%
		65–74	0.65%	2.84%	4.12%	6.73%	9.23%
		>=75	0.68%	2.06%	2.78%	4.23%	5.48%
	Total		0.86%	3.44%	4.89%	8.04%	11.39%

The difference in QALE between both scenarios represents the incremental HRQoL associated with using an implant with lower revision risk. By comparing these HRQoL gains with the additional costs associated with the implant, the ICER was estimated. The only differential cost included in the analysis was the implant acquisition cost. WorstKnee was assigned the lowest price identified in a public tender of the Spanish Health System (€1,100) [30], whereas BestKnee was assigned the highest reported price (€2,574) [31].

In summary, the model applies published age- and sex-specific parameters to a real-world patient cohort with known baseline utilities. This allows individualized long-term projections of HRQoL, life expectancy, and prosthetic failure, providing a transparent framework for evaluating the clinical and economic impact of different knee implant models.

A 15-year simulation horizon was selected because this is the longest period for which reliable implant survival data are currently available [29, 32]. Furthermore, given that TKA is intended as a long-term treatment, shorter time horizons would not allow an adequate assessment of implant performance and long-term cost-effectiveness.

### Real-world data

As stated previously, the model was parameterized using real-world patient-level utility data obtained from a prospective multicenter cohort of patients undergoing primary TKA. The data collected included demographic (age and sex), and quality-of-life (SF-12v2 [33]) variables.

All subjects were evaluated preoperatively and one year after surgery; patients lost to follow-up were excluded from the analysis. Three-year postoperative HRQoL data were used for model validation.

The index used to calculate the QALYs for each treatment alternative was the Short Form 6D (SF-6D), derived from the SF-12v2 questionnaire using the method proposed by Brazier et al. [34, 35]. Final utility values were calculated using Spanish population preferences according to the value set developed by Martínez-Pérez et al. [36].

The study was approved by the Research Ethics Committee of Aragón (reference number PI25/236).

### Sources of information

The sources of information incorporated into the model are summarized in Table 2, whereas the survival of the compared implant strategies was estimated using the age- and sex-stratified revision rates shown in Table 1.

**Table 2:** Sources of information for the model, budgetary and sensitivity analyses

General model		
Model component	Description	Source
Patient-level clinical data	Age and sex of the patients Preoperative SF-12v2 utility values 1-year postoperative SF-12v2 utility values	Multicenter real-world cohort
Utility calculation	Conversion of SF-12v2 scores into SF-6D utility values	Brazier (2004) [34]
Population utility preferences	Spanish population utility weights used to calculate SF-6D values	Martínez-Pérez [36]
Mortality	Age- and sex-specific mortality risks	Spanish Ministry of Health (2021) [28]
Postoperative recovery	Assumption of full recovery one year after surgery	Elmallah (2017) [26] and Ethgen (2004) [3]
Implant survival	Age- and sex-specific revision rates applied in the simulation	UK National Joint Registry (2022) [29]
Quality of life evolution	Age- and sex-specific decline in quality of life over time	Van den Berg (2012) [27]
Implant costs	Acquisition cost of each implant strategy	Public tenders SNS [30, 31]
Willingness-to-pay threshold	WTP used for ICER interpretation	Sacristán (2020) [37] and NICE [38]
Budget impact analysis		
Model component	Description	Source
Methodology	General calculation framework	Spanish Ministry of Health (2014) [39] and Brosa (2005) [40]
Primary TKA surgeries	National volume of primary surgeries by year	Spanish Ministry of Health (2024) [9]
Revision surgery costs	Estimated cost of revision procedures	Gungor (2020) [41]
Discount rate	Annual discount rate applied to the budget impact analysis	Spanish Ministry of Health (2023) [25]
Sensitivity analyses		
Model component	Description	Source
SA-1	WorstKnee associated with higher revision rates	Australian National Joint Registry (2022) [32]
SA-2	BestKnee associated with higher revision rates	-
SA-3	WorstKnee associated with lower revision rates	-
SA-4	Greater impact of implant failure on quality of life	-
SA-5	5% annual discount rate	Spanish Ministry of Health (2023) [25]

### Validation of the model

An operational validation of the model was performed following previously published recommendations [42, 43]. Given that the proposed model assumes that postoperative HRQoL evolves according to the natural age- and sex-specific trajectories of the general population, rather than remaining stable over time and being adjusted solely through fixed annual discounting, its predictions under the assumption of no implant failure were compared with real postoperative outcomes.

Specifically, the three-year HRQoL values predicted by the discrete-event simulation model were compared with the actual three-year postoperative outcomes observed in patients with available follow-up data (*N* = 186).

Predictions were also compared with those obtained using conventional approaches based on annual discount rates of 3% and 5%, as commonly recommended in economic evaluations [25].

Although statistically significant differences were observed in all approaches, these differences were substantially smaller with the discrete-event simulation model (Cohen’s d: 0.1096 vs. 0.3137 vs. 0.5164), with a negligible effect size according to Cohen’s interpretation criteria [39].

**Fig. 3:**
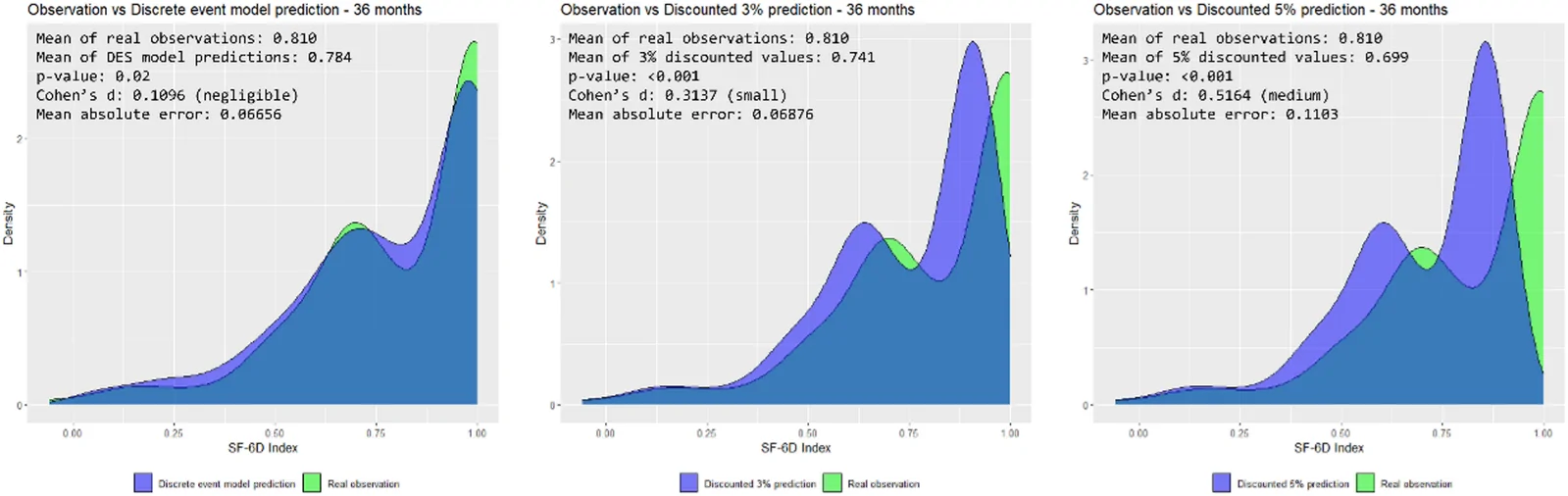
Comparison between the three approaches used to project postoperative HRQoL (discrete-event simulation model, 3% annual discounting, and 5% annual discounting) and the actual HRQoL observed at 3-year follow-up in the clinical cohort

These findings provide preliminary support for the plausibility of modeling postoperative HRQoL according to age- and sex-adjusted population trajectories rather than approaches based solely on fixed annual discounting (Fig. 3).

### Discount rates

No cost discounting was required in the incremental analysis, as these costs are incurred at the time of treatment. A 3% discount rate was used for the multi-annual budgetary impact analysis. A sensitivity analysis was also performed, using a 5% discount rate [25].

Regarding health outcomes, no additional exogenous discount rate was applied, as the model already adjusts quality of life according to the expected age- and sex-specific deterioration over time [27], while also restricting outcomes according to each patient’s life expectancy [28].

### Statistical analysis and decision-making criteria

Descriptive analyses were performed using measures of central tendency and dispersion. Differences between treatment alternatives were evaluated using Student’s t test. Statistical significance was set at *p* < 0.05. The incremental cost-effectiveness ratio (ICER) was calculated [24, 40] and compared with the cost-effectiveness thresholds commonly used in Spain [37, 38, 44]. Subgroup analyses were performed by age group, following the classifications used in major international registries [29, 32].

Data were analyzed using R software (R Development Core Team), version 4.5.2 [45].

### Budget impact analysis

The budget impact analysis was conducted following a methodology proposed by the Spanish Ministry of Health [46]. Revision rates for each treatment alternative were applied to the annual number of primary TKA performed within the Spanish National Health System (39,487 procedures/year) [9], to estimate the number of revision surgeries expected if all patients received either the BestKnee or the WorstKnee implant.

Based on the expected number of primary and revision procedures associated with each implant alternative, the required operating room days and the corresponding costs for the Spanish National Health System were estimated.

As it was the only differential cost between the two treatment alternatives, prosthesis cost was the only cost considered in the discrete-event model. In the budget impact analysis, however, revision surgeries also needed to be considered, given that each alternative has different implications for the expected number of revision procedures. Revision surgeries were incorporated assuming a cost three times higher than that of primary surgery [41].

Operating room requirements were estimated according to the productivity assumption, whereby a single operating room day can accommodate either three primary procedures or one primary and one revision procedure, but not two primary and one revision procedure [46].

Budget impact was calculated using the conventional formula for budget impact analyses [47, 48], applying annual discount rates of 3% and 5% [25]. The complete model and all modifiable parameters are provided in Appendix 1.

$$\:Budget\:impact=\left({CM}_{B}-{CM}_{A}\right)\cdot\:N$$

where A is the existing technology and B is the new technology:

$$\:{CM}_{A}:Mean\:cost\:per\:patient\:treated\:by\:A$$

$$\:{CM}_{B}:Mean\:cost\:per\:patient\:treated\:by\:B$$

$$\:N:Number\:of\:patients$$

### Deterministic sensitivity analysis

A deterministic sensitivity analysis was performed to assess the impact of key model parameters on cost-effectiveness outcomes and budget impact. Four scenarios were evaluated:

- SA-1: WorstKnee associated with higher revision rates. This analysis was based on a very poorly performing cohort reported in the Australian Orthopaedic Association National Joint Replacement Registry (AOANJR), with a 15-year revision rate of 21.2% [32].
- SA-2: BestKnee associated with higher revision rates. This scenario assumes a 25% decrease in survival at each follow-up timepoint.
- SA-3: WorstKnee associated with lower revision rates. This scenario assumes a 25% improvement in survival at each follow-up timepoint.
- SA-4: Prosthetic failure was assumed to have a stronger impact on HRQoL [49–52]. Implant failure results in a HRQoL drop below the preoperative level, specifically to a value halfway between the minimum expected HRQoL and death.

## Results

### Characteristics of the cohort used in the simulation

The simulation was performed on a real-world cohort derived from an initial database of 970 individuals. After application of the predefined exclusion criteria, the final cohort included 486 patients, comprising 343 (70.6%) women and 143 (29.4%) men, with a mean age of 71.9 ± 7.6 years (Table 3).

**Table 3:** Origin and characteristics of the cohort used in the simulation

Contributing center	N	Mean age (years)	Sex (M/F)	PreOp HRQoL	1-year HRQoL
San Agustín University Hospital (Avilés)	37	70.8	11/26	0.281	0.685
Lozano Blesa Clinical University Hospital (Zaragoza)	84	72.5	24/60	0.333	0.643
Miguel Servet University Hospital (Zaragoza)	30	70.2	12/18	0.448	0.835
San Juan Grande Hospital (Cádiz)	165	70.8	48/117	0.103	0.957
Valladolid Clinical University Hospital	19	74.2	2/17	0.425	0.560
Virgen del Puerto Hospital	151	73.0	46/105	0.369	0.669
**TOTAL OR MEAN**	**486**	**71.9**	**143/343**	**0.273**	**0.770**

### Health outcomes

**Fig. 4:**
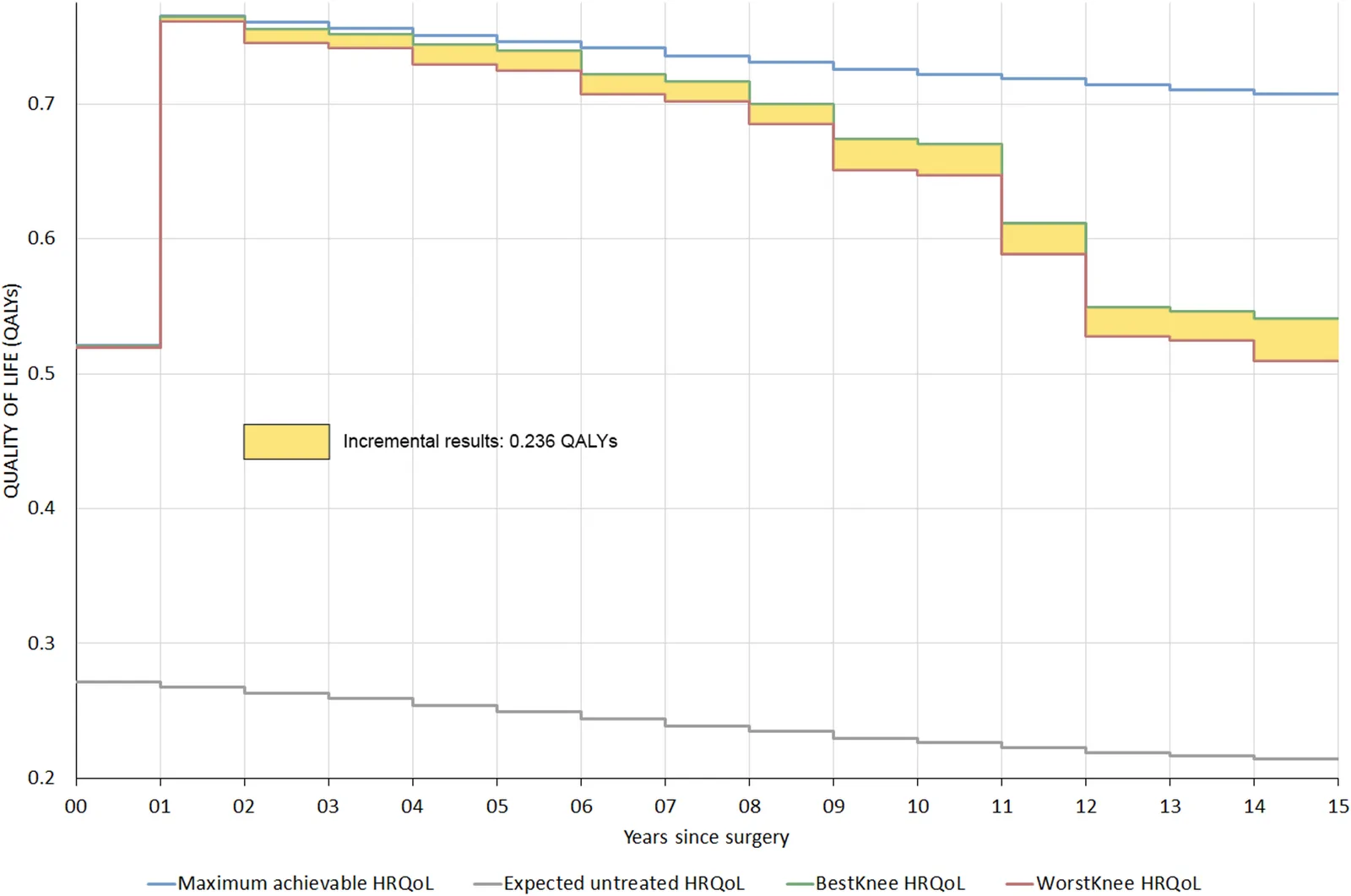
Mean number of QALYs obtained by the patients in the model when treated with the BestKnee (green) and the WorstKnee (red). The yellow area reflects the difference in health outcomes between the two treatment alternatives

Using the assumptions described in the Materials and Methods section, the maximum achievable and expected untreated HRQoL trajectories were projected for each individual in the cohort, together with the expected HRQoL outcomes associated with each implant alternative. The resulting estimates are summarized in Table 4 and represented in Fig. 4.

Mean quality-adjusted life expectancy was estimated at 9.767 QALYs for WorstKnee and 10.004 QALYs for BestKnee (*p* < 0.001).

**Table 4:** Health outcomes according to the model (quality of life as measured by the SF-6D index)

Year	Maximum achievable HRQoL	Expected untreated HRQoL	BestKnee HRQoL	WorstKnee HRQoL
1	0.521	0.272	0.521	0.519
2	0.766	0.268	0.764	0.762
3	0.760	0.263	0.755	0.745
4	0.756	0.259	0.751	0.741
5	0.751	0.254	0.744	0.730
6	0.746	0.249	0.739	0.725
7	0.741	0.244	0.722	0.708
8	0.736	0.239	0.716	0.702
9	0.731	0.235	0.699	0.686
10	0.726	0.230	0.674	0.651
11	0.722	0.226	0.670	0.647
12	0.719	0.223	0.611	0.589
13	0.714	0.219	0.549	0.528
14	0.711	0.217	0.546	0.525
15	0.707	0.214	0.541	0.510
**Total**	**10.808**	**3.609**	**10.004**	**9.767**

This resulted in an ICER of €6,219/QALY.

$$\begin{aligned}\:ICER\;&=\;\frac{\triangle C}{\triangle R}\;=\;\frac{{}^CBestKnee{}^{-C}WorstKnee}{{}^R{BestKnee{}^{-R}{WorstKnee}}}\\&=\frac{\mathrm{\text\euro}2,574\;-{{\text\euro}}1,100}{10.004\;QALYs-9.767\;QALYs}\\&\approx\mathrm{\text\euro}6,219/QALY\end{aligned}$$

To evaluate the cost-effectiveness of the two treatment alternatives, the estimated ICER was compared with the cost-effectiveness thresholds most commonly reported in the literature [37, 44]. The resulting ICER remained below all published reference thresholds. Assuming a conservative willingness-to-pay threshold of €25,000/QALY [37], the maximum acceptable price for the BestKnee would be €7,025.

$$\begin{aligned}\:\mathrm{\text\euro}\mathrm{25,000}&=\frac{Maximum\:price-\mathrm{\text\euro}\mathrm{1,100}}{10.004\:QALYs-9.767\:QALYs}\\&\to\:Maximum\:price=\mathrm{\text\euro}\mathrm{7,025}\end{aligned}$$

### Results by age group

Mean ICER values were €1,800/QALY for patients younger than 55 years, €3,649/QALY for those aged 55–64 years, €5,321/QALY for those aged 65–74 years, and €14,311/QALY for patients older than 75 years. These findings indicate that implants with greater survivorship are comparatively more cost-effective in younger patients, although ICER values remained below commonly accepted cost-effectiveness thresholds across all age groups.

### Budget impact analysis

Based on the model projections, the expected number of revision surgeries during the 15-year study period was estimated [46]. Compared with WorstKnee, BestKnee was associated with 27,878 fewer revision procedures, corresponding to 18,585 fewer operating room days over the study period.

**Fig. 5:**
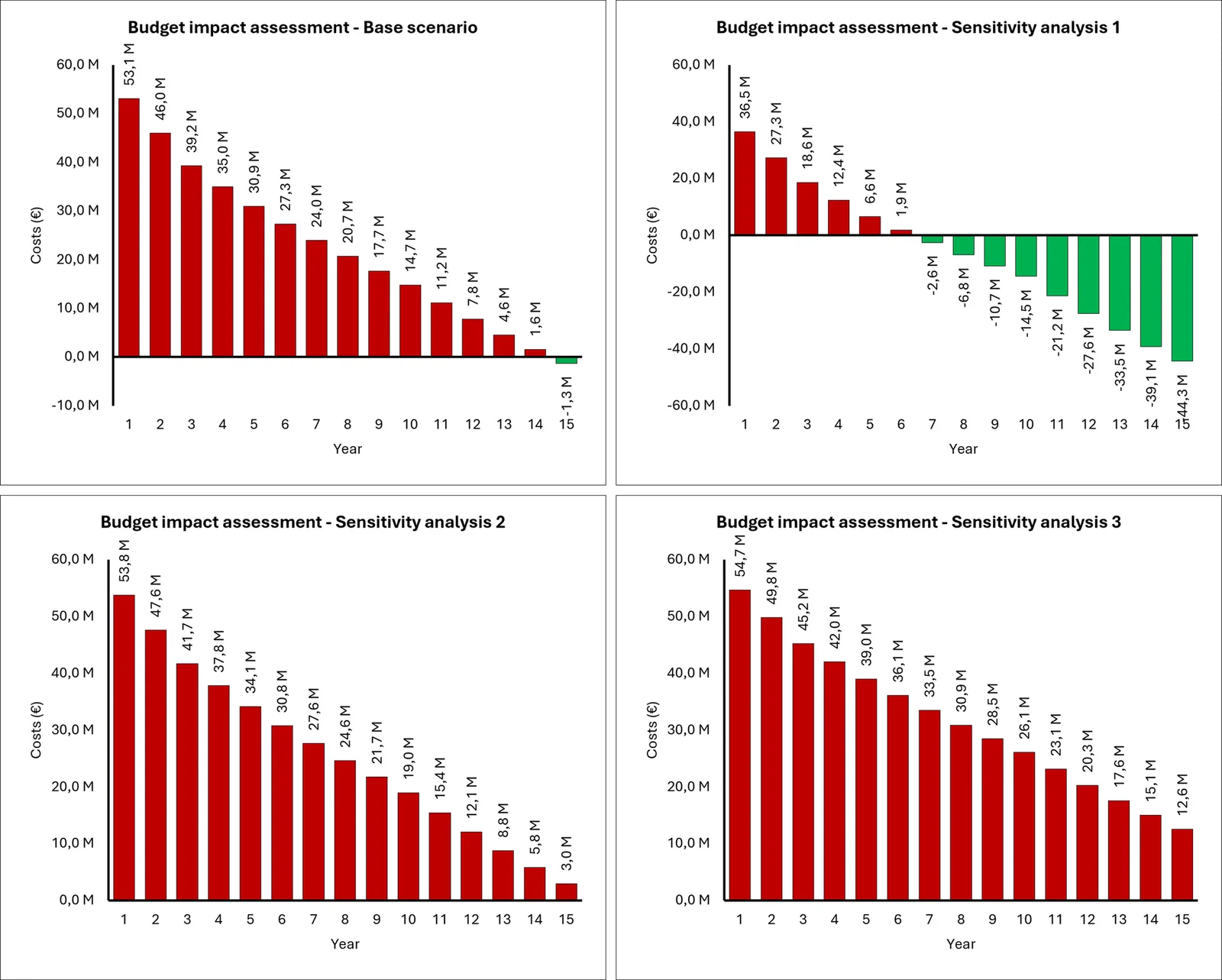
Budget impact of the baseline scenario and of the various sensitivity analyses. Red bars: cost increases resulting from the introduction of the BestKnee; green bars, cost reductions

Applying a 3% annual discount rate, introduction of BestKnee would result in an additional expenditure of €332.3 M over 15 years. With a 5% discount rate, the additional expenditure would decrease to €302.9 M. However, these additional costs progressively declined over time as a result of the lower revision burden associated with BestKnee, with net savings potentially emerging during the final year of the study period (Table 5 and Fig. 5).

**Table 5:** Budgetary analysis – baseline scenario

Year	BestKnee			WorstKnee			Updated cost difference [WorstKnee – BestKnee]
	Updated cost of primary surgeries	Updated cost of revision surgeries	Updated total cost	Updated cost of primary surgeries	Updated cost of revision surgeries	Updated total cost	
1	€302,861,456	€2,899,223	€305,760,680	€246,352,876	€6,344,895	€252,697,771	€53,062,908
2	€294,040,249	€6,523,141	€300,563,390	€239,177,549	€15,427,488	€254,605,037	€45,958,352
3	€285,475,970	€9,933,497	€295,409,466	€232,211,213	€23,957,973	€256,169,186	€39,240,280
4	€277,161,136	€11,434,029	€288,595,164	€225,447,779	€28,175,977	€253,623,756	€34,971,408
5	€269,088,481	€12,838,724	€281,927,205	€218,881,339	€32,111,318	€250,992,657	€30,934,548
6	€261,250,953	€13,794,622	€275,045,575	€212,506,155	€35,196,147	€247,702,302	€27,343,273
7	€253,641,702	€14,683,945	€268,325,646	€206,316,655	€38,058,361	€244,375,016	€23,950,630
8	€246,254,079	€15,509,760	€261,763,839	€200,307,432	€40,739,205	€241,046,637	€20,717,202
9	€239,081,630	€16,293,177	€255,374,807	€194,473,235	€43,231,597	€237,704,832	€17,669,975
10	€232,118,088	€16,982,530	€249,100,618	€188,808,966	€45,544,240	€234,353,206	€14,747,411
11	€225,357,367	€17,018,656	€242,376,023	€183,309,676	€47,894,394	€231,204,069	€11,171,953
12	€218,793,560	€17,038,271	€235,831,831	€177,970,559	€50,082,530	€228,053,089	€7,778,741
13	€212,420,932	€17,042,305	€229,463,237	€172,786,950	€52,102,571	€224,889,521	€4,573,715
14	€206,233,915	€17,031,651	€223,265,565	€167,754,321	€53,949,708	€221,704,029	€1,561,536
15	€200,227,101	€16,991,947	€217,219,049	€162,868,273	€55,645,044	€218,513,317	-€1,294,268
**TOTAL**	**€3,724,006,618**	**€206,015,477**	**€3,930,022,095**	**€3,029,172,978**	**€568,461,447**	**€3,597,634,425**	**€332,387,669**

### Sensitivity analysis

#### SA-1: WorstKnee associated with higher revision rates

Using the worst revision rates identified in the literature (AOANJR), projected QALE for WorstKnee decreased to 9.477 QALYs over 15 years, resulting in an ICER of €2,800/QALY for BestKnee versus WorstKnee. Under this scenario, use of BestKnee would avoid 57,294 revision procedures and reduce operating room utilization by 38,196 days over the study period. In this context, adoption of BestKnee would not increase expenditure but instead generate savings of €97.1 M over 15 years (Fig. 5).

#### SA-2: BestKnee associated with higher revision rates

Assuming revision rates for BestKnee were 25% higher than published, projected QALE decreased to 9.977 QALYs. As outcomes for WorstKnee remained unchanged, the resulting ICER increased to €7,012/QALY. Under this scenario, introduction of BestKnee would avoid 25,009 revision procedures and reduce operating room utilization by 16,674 days over 15 years. The additional cost associated with BestKnee would amount to €383.9 M over the study period.

#### SA-3: WorstKnee associated with lower revision rates

Assuming revision rates for WorstKnee were 25% lower than observed, projected QALE increased to 9.852 QALYs over 15 years. As outcomes for BestKnee remained unchanged, the resulting ICER increased to €9,739/QALY. Under this scenario, introduction of BestKnee would avoid 18,041 revision procedures and reduce operating room utilization by 12,028 days. The associated additional expenditure would amount to €474.5 M over the 15-year study period.

#### SA-4: Prosthetic failure associated with a greater impact on HRQoL

Assuming that prosthetic failure had a stronger impact on HRQoL, with utility decreasing below the preoperative level to a value halfway between the minimum expected HRQoL and death, projected QALE decreased to 9.980 QALYs for BestKnee and 9.691 QALYs for WorstKnee over 15 years. The resulting ICER was €5,102/QALY for BestKnee versus WorstKnee.

## Discussion

The main finding of this study was that, within the price ranges currently observed in the Spanish National Health System, and under the assumption that implant price is associated with implant performance, total knee prostheses with lower revision rates appear to provide a more favorable long-term cost-effectiveness profile despite their higher acquisition cost.

### Interpretation of results

The estimated ICER associated with adoption of BestKnee ranged from €2,800/QALY to €9,739/QALY across the different sensitivity scenarios considered. Although no official cost-effectiveness threshold has been established in Spain, the values most commonly cited in the literature range between €20,000/QALY and €30,000/QALY [37, 44, 53, 54]. In all scenarios analyzed, the ICER estimated by the model remained well below these reference thresholds, supporting the use of implants associated with lower revision rates even at moderately higher acquisition costs and across all age groups considered.

These findings are in line with those reported by Pennington et al. [23], the only previous study comparing different prosthetic models. In that analysis, the ICER between the most and the least cost-effective prosthesis was estimated at £2,300/QALY (currently €3,650/QALY). The difference between their estimate and ours is relatively small and may be explained by the prostheses included in each comparison. While our analysis contrasted implants with markedly different revision profiles, their study evaluated the five leading knee prostheses on the market, which were associated with more similar long-term outcomes and therefore smaller incremental differences.

### Rationale of the model

Although Spanish legislation identifies cost-effectiveness analyses as one of the criteria supporting healthcare funding decisions [55], their application to arthroplasties remains challenging. The parameters that define the quality of TKA are not clearly defined and there are no randomized clinical trials comparing the performance of the different options available, with survival remaining as the main comparison variable [15]. However, demonstrating an implant’s survival requires long-term follow-ups, which could defer the introduction of new technologies. In this context, and given the absence of a nationwide arthroplasty registry in Spain [56], simulation models currently represent one of the most useful approaches for evaluating the long-term economic impact of different implants [10, 21].

Discrete-event simulation models analyze systems whose status changes at discrete time intervals, impacting patient health or resource consumption [56, 57]. Importantly, the probability of these events can be individualized according to each patient’s characteristics and risk profile, allowing patient-specific HRQoL trajectories to be modeled [57, 58]. We therefore considered this methodology particularly appropriate for the present analysis.

The methodology used in this study is based on two studies by Elmallah et al. [26, 59] and on a report by the Spanish Ministry of Health [46].

Elmallah measured HRQoL in patients undergoing TKA preoperatively and one year after surgery. Using these two timepoints as a basis, and assuming that postoperative HRQoL would not decline below baseline values, they projected HRQoL forward in time. Using mean life expectancy estimates, they then calculated the cost-effectiveness of TKA from the difference in QALYs between two alternatives: one in which patients underwent surgery and another in which preoperative HRQoL was projected over time as an untreated scenario. The present study builds upon and improves this approach, incorporating the natural HRQoL reduction experienced by subjects over time, specific to their age and sex [27]. Moreover, our model includes the possibility of prosthetic failure, using revision data from official registries stratified by age and sex, together with individualized life expectancy estimates, also stratified by age and sex, for every subject in the cohort.

In addition, the report published by the Spanish Ministry of Health [46] describes a method for estimating the surgical burden imposed on the Spanish National Health System based on the effect of prosthetic revision rates on the expected volume of surgical procedures over time. This approach was applied to the present analysis, incorporating as a novel element a differentiated economic valuation of primary and revision procedures, followed by a conventional budget impact analysis [47].

### Inclusion of revision surgeries in the analysis

Incorporation of revision procedures into the model as a major cost component is particularly relevant, as the number of revision surgeries is expected to increase in parallel with the growing volume of primary TKA procedures [3]. Revision arthroplasties are considerably more complex than primary procedures, often requiring implant removal, management of bone loss, larger surgical exposures, and are associated with higher complication and infection rates [19]. As a result, hospital stay following revision TKA may be up to twice that observed after primary surgery, while associated costs may exceed those of primary procedures by more than threefold [41]. Importantly, the economic burden associated with TKA is not determined exclusively by implant acquisition costs, but also by revision burden, operating room occupation, hospital stay, complications, rehabilitation requirements, and the progressive increase in procedural volume driven by population ageing.

The reduction observed in the number of revision surgeries when implants with a longer lifespan were used resulted in the model showing only a moderately higher expenditure (€22 M on average per year). It must be noted that this higher expenditure decreased progressively over time, eventually leading to net savings associated with use of the BestKnee (Fig. 5).

Another beneficial effect of potentially decreasing the number of revisions is the reduction of waiting-lists, which have become a problem for many OECD countries, exacerbated by the impact of the COVID pandemic. Postponement of the benefits expected from treatment condemns patients to experience pain and disabilities for longer than necessary, which reduces their quality of life and may even negatively impact the outcome of their revision procedure [49–52].

### The situation in Spain

Health systems should reduce costs without compromising quality [10, 11]. In the case of arthroplasties, implant selection plays a major role in clinical outcomes [11, 14], so strategies focused exclusively on reducing implant prices may result in worse results [20]. Fortunately, alternative approaches do exist. Hospitals may avoid negative impacts and simultaneously attain their cost and quality targets by redesigning their clinical and administrative procedures [60] and implementing fast-track rehabilitation programs, which have been shown to shorten the hospital stay and improve clinical outcomes [61].

In Spain, the most common procurement model is based on public tenders combining quality and economic criteria, allowing a limited number of suppliers to cover the demand of a hospital or regional healthcare system through framework agreements. Although this strategy has likely contributed to a substantial reduction in implant prices, its potential unintended consequences should also be considered. Because the Spanish National Health System effectively operates as a demand oligopoly, it may exert sufficient pricing pressure to discourage investment in these products. Indeed, the current Spanish prosthetic market could be described as one of increasing procedural volume but progressively narrowing profit margins, potentially threatening the sustainability of supplier companies. Rapid and sustained price reductions may artificially accelerate product life cycles and reduce the attractiveness of conventional primary arthroplasty systems for manufacturers, potentially shifting investment away from incremental innovation in these implants towards higher-value market segments such as robotics or personalized prosthetic technologies.

Nevertheless, it should be acknowledged that the economic assumptions incorporated into the model were developed from the perspective of the Spanish National Health System and may therefore not be directly transferable to private healthcare environments or to healthcare systems with different procurement, reimbursement, and registry structures. In addition, the present model did not attempt to reproduce implant selection policies at the individual hospital level. These considerations may differ from those observed in other publicly funded healthcare systems such as the British or Scandinavian models, where nationwide arthroplasty registries are more firmly integrated into procurement policies and healthcare planning. Beyond implant surveillance, nationwide arthroplasty registries may contribute substantially to healthcare sustainability by enabling cost-effectiveness analyses based on real-world long-term survivorship data.

### Strengths and limitations of the study

One of the main strengths of this study lies in the use of a discrete-event simulation model based on a real multicenter clinical cohort, which allowed the incorporation of individualized quality-of-life outcomes obtained from routine clinical practice rather than relying exclusively on average values extracted from the literature. This approach made it possible to model patient-specific trajectories according to age and sex, perform subgroup analyses, and generate estimates that may better reflect real-world clinical practice within the Spanish National Health System.

As far as the study design is concerned, it should be noted that the population analyzed was not extracted from a randomized clinical trial. It would be ethically unacceptable to treat two groups of patients with different prostheses knowing in advance that their performance will be different. However, the fact that subjects were extracted from a multicenter observational series is associated with two positive effects: firstly, the study is representative of routine clinical practice (which is not always the case when subjects are drawn from a clinical trial), and secondly it reflects the heterogeneity that characterizes a country like Spain as it includes patients from five different regions. Nevertheless, although the cohort included hospitals from different geographical areas, the limited territorial coverage may restrict the full generalizability of the findings to the entire Spanish population.

The timeframe selected for the study could also elicit some criticism, but in the absence of previous registries and given that the few knees that have been followed up for 20 years or more exhibit only mildly increasing annual revision risk over time [29, 32], a longer-term analysis should attest to even greater differences in favor of the longer-surviving implant. However, this assumption cannot be confirmed based on the currently available data. As with any long-term simulation model, the results are partly dependent on the structural assumptions and extrapolation methods incorporated into the analysis.

It should also be noted that the survival data included were extracted from the UK [29] and Australian [32] registries, as there is no nationwide registry in Spain and no reports have been published anywhere in the European Union that show significant differences in revision rates after TKA. Similarly, in the absence of Spanish population norms for the SF-6D index, UK population reference values were used to model the age- and sex-specific evolution of HRQoL over time [27]. It must nevertheless be acknowledged that the use of parameters derived from sources with different geographical origins may introduce a certain degree of heterogeneity into the model and could potentially influence the external validity of the estimates obtained.

It must be recognized that a model where sex and age are the only factors determining prosthetic survival ignores other potentially significant variables such as obesity, the patient’s activity level or implant design (e.g., posterior cruciate ligament preserving vs. sacrificing prostheses). Nonetheless, it must be noted that not enough disaggregated data is available to include such variables, and that their actual impact on implant longevity remains unknown.

The last limitation to be mentioned is related to the fact that the main difference between the analyzed implants is their survival, which depends only on the implants themselves, and that the implants’ price only depends on their performance. In this connection, it is widely known that the prosthetic model selected is the factor that most directly influences the results of TKA [14], that the revision rate is the chief indicator of an implant’s quality [15] and that, as shown by previous studies, there is a strong reverse correlation between an implant’s revision rate and its price [23](*r*=-0.961, *p* = 0.009), which would seem to indicate that correlating both parameters is a reasonable approach.

Despite these limitations, we believe the present study may be of interest to the scientific community, as it represents only the second study worldwide comparing different knee prosthetic models and the first to perform an individualized discrete-event simulation based on a multicenter Spanish cohort, incorporating real-world baseline HRQoL data together with age- and sex-specific projections of mortality, quality of life, and prosthetic revision risk to evaluate cost-effectiveness and budget impact.

## Conclusion

Within the price ranges currently observed in the Spanish National Health System, total knee prostheses associated with lower revision rates provide a favorable long-term cost-effectiveness profile and may reduce future revision burden despite their higher acquisition cost.

## Supplementary Information

Supplementary Material 1.

Supplementary Material 2.

## Data Availability

The datasets generated and/or analysed during the current study are not publicly available due to their medical nature but are available from the corresponding author on reasonable request.

## Abbreviations

TKA: Total knee arthroplasty
ICD-9-CM: International Classification of Diseases, Ninth Revision, Clinical Modification
ICD-10-PCS: International Classification of Diseases, Tenth Revision, Procedure Coding System
QALYs: Quality-adjusted life years
SF-12v2: Short Form 12 version 2
SF-6D: Short Form 6D
NJR: United Kingdom’s National Joint Registry
ICER: Incremental cost-effectiveness ratio
AOANJR: Australian Orthopaedic Association National Joint Replacement Registry
QALE: Quality-adjusted life expectancy
HRQoL: Health-related quality of life
