# Supplementary material for: The influence of implant survival on cost-effectiveness in total knee arthroplasty: a patient-level discrete-event simulation study
Source: Health Econ Rev. 2026 Jul 17;16:126. doi: 10.1186/s13561-026-00822-z (PMC13711803; doi:10.1186/s13561-026-00822-z)
Supplement: Supplementary file 1 [file 13561_2026_822_MOESM2_ESM.pdf]

CHEERS 2022 Checklist

	Item	Guidance for Reporting	Reported in section
**TITLE**			
Title	1	Identify the study as an economic evaluation and specify the interventions being compared.	Title
**ABSTRACT**			
Abstract	2	Provide a structured summary that highlights context, key methods, results and alternative analyses.	Abstract
**INTRODUCTION**			
Background and objectives	3	Give the context for the study, the study question and its practical relevance for decision making in policy or practice.	Introduction
**METHODS**			
Health economic analysis plan	4	Indicate whether a health economic analysis plan was developed and where available.	Not applicable
Study population	5	Describe characteristics of the study population (such as age range, demographics, socioeconomic, or clinical characteristics).	Materials and Methods – Real-world data; Results – Characteristics of the cohort used in the simulation
Setting and location	6	Provide relevant contextual information that may influence findings.	Introduction; Materials and Methods – Real-world data
Comparators	7	Describe the interventions or strategies being compared and why chosen.	Materials and Methods – Rationale of the model
Perspective	8	State the perspective(s) adopted by the study and why chosen.	Materials and Methods – Rationale of the model
Time horizon	9	State the time horizon for the study and why appropriate.	Materials and Methods – Rationale of the model
Discount rate	10	Report the discount rate(s) and reason chosen.	Materials and Methods – Budget impact analysis; Materials and Methods – Deterministic sensitivity analysis
Selection of outcomes	11	Describe what outcomes were used as the measure(s) of benefit(s) and harm(s).	Materials and Methods – Objectives
Measurement of outcomes	12	Describe how outcomes used to capture benefit(s) and harm(s) were measured.	Materials and Methods – Real-world data; Materials and Methods – Rationale of the model
Valuation of outcomes	13	Describe the population and methods used to measure and value outcomes.	Materials and Methods – Real-world data
Measurement and valuation of resources and costs	14	Describe how costs were valued.	Materials and Methods – Budget impact analysis
Currency, price date, and conversion	15	Report the dates of the estimated resource quantities and unit costs, plus the currency and year of conversion.	Materials and Methods – Budget impact analysis
Rationale and description of model	16	If modelling is used, describe in detail and why used. Report if the model is publicly available and where it can be accessed.	Materials and Methods – Rationale of the model; Figure 1; Figure 2
Analytics and assumptions	17	Describe any methods for analysing or statistically transforming data, any extrapolation methods, and approaches for validating any model used.	Materials and Methods – Rationale of the model; Materials and Methods – Deterministic sensitivity analysis; Materials and Methods – Model validation
Characterizing heterogeneity	18	Describe any methods used for estimating how the results of the study vary for sub-groups.	Materials and Methods – Deterministic sensitivity analysis; Discussion – Strengths and limitations
Characterizing distributional effects	19	Describe how impacts are distributed across different individuals or adjustments made to reflect priority populations.	Not applicable
Characterizing uncertainty	20	Describe methods to characterize any sources of uncertainty in the analysis.	Materials and Methods – Deterministic sensitivity analysis; Results – Sensitivity analysis
Approach to engagement with patients and others affected by the study	21	Describe any approaches to engage patients or service recipients, the general public, communities, or stakeholders (e.g., clinicians or payers) in the design of the study.	Not applicable
**RESULTS**			
Study parameters	22	Report all analytic inputs (e.g., values, ranges, references) including uncertainty or distributional assumptions.	Table 2; Materials and Methods
Summary of main results	23	Report the mean values for the main categories of costs and outcomes of interest and summarise them in the most appropriate overall measure.	Results
Effect of uncertainty	24	Describe how uncertainty about analytic judgments, inputs, or projections affect findings. Report the effect of choice of discount rate and time horizon, if applicable.	Results – Sensitivity analysis; Discussion
Effect of engagement with patients and others affected by the study	25	Report on any difference patient/service recipient, general public, community, or stakeholder involvement made to the approach or findings of the study	Not applicable
**DISCUSSION**			
Study findings, limitations, generalizability, and current knowledge	26	Report key findings, limitations, ethical or equity considerations not captured, and how these could impact patients, policy, or practice.	Discussion – Strengths and limitations; Conclusion
**OTHER RELEVANT INFORMATION**			
Source of funding	27	Describe how the study was funded and any role of the funder in the identification, design, conduct, and reporting of the analysis	Funding
Conflicts of interest	28	Report authors conflicts of interest according to journal or International Committee of Medical Journal Editors requirements.	Conflicts of interest / Declarations

Husereau D, Drummond M, Augustovski F, de Bekker-Grob E, Briggs AH, Carswell C, Caulley L, Chaiyakunapruk N, Greenberg D, Loder E, Mauskopf J, Mullins CD, Petrou S, Pwu RF, Staniszewska S; CHEERS 2022 ISPOR Good Research Practices Task Force. Consolidated Health Economic Evaluation Reporting Standards 2022 (CHEERS 2022) Statement: Updated Reporting Guidance for Health Economic Evaluations. BMJ. 2022;376:e067975.
The checklist is Open Access distributed in accordance with the terms of the Creative Commons Attribution (CC BY 4.0) license, which permits others to distribute, remix, adapt and build upon this work, for commercial use, provided the original work is properly cited. See: http://creativecommons.org/licenses/by/4.0/.
